# Effective cerebello–cerebral connectivity during implicit and explicit social belief sequence learning using dynamic causal modeling

**DOI:** 10.1093/scan/nsac044

**Published:** 2022-07-07

**Authors:** Qianying Ma, Min Pu, Naem Haihambo, Kris Baetens, Elien Heleven, Natacha Deroost, Chris Baeken, Frank Van Overwalle

**Affiliations:** Department of Psychology, Center for Neuroscience, Vrije Universiteit Brussel, Brussels 1050, Belgium; Department of Psychology, Center for Neuroscience, Vrije Universiteit Brussel, Brussels 1050, Belgium; Department of Psychology, Center for Neuroscience, Vrije Universiteit Brussel, Brussels 1050, Belgium; Department of Psychology, Center for Neuroscience, Vrije Universiteit Brussel, Brussels 1050, Belgium; Department of Psychology, Center for Neuroscience, Vrije Universiteit Brussel, Brussels 1050, Belgium; Department of Psychology, Center for Neuroscience, Vrije Universiteit Brussel, Brussels 1050, Belgium; Faculty of Medicine and Health Sciences, Department of Head and Skin, Ghent Experimental Psychiatry (GHEP) Lab, Ghent Experimental, Ghent University, Ghent 9000, Belgium; Department of Psychiatry, University Hospital (UZBrussel), Brussels 1090, Belgium; Department of Electrical Engineering, Eindhoven University of Technology, Eindhoven 5600, The Netherlands; Department of Psychology, Center for Neuroscience, Vrije Universiteit Brussel, Brussels 1050, Belgium

**Keywords:** cerebellum, serial reaction time task, dynamic causal modeling, social cognition, false belief

## Abstract

To study social sequence learning, earlier functional magnetic resonance imaging (fMRI) studies investigated the neural correlates of a novel Belief Serial Reaction Time task in which participants learned sequences of beliefs held by protagonists. The results demonstrated the involvement of the mentalizing network in the posterior cerebellum and cerebral areas (e.g. temporoparietal junction, precuneus and temporal pole) during implicit and explicit social sequence learning. However, little is known about the neural functional interaction between these areas during this task. Dynamic causal modeling analyses for both implicit and explicit belief sequence learning revealed that the posterior cerebellar Crus I & II were effectively connected to cerebral mentalizing areas, especially the bilateral temporoparietal junction, via closed loops (i.e. bidirectional functional connections that initiate and terminate at the same cerebellar and cerebral areas). There were more closed loops during implicit than explicit learning, which may indicate that the posterior cerebellum may be more involved in implicitly learning sequential social information. Our analysis supports the general view that the posterior cerebellum receives incoming signals from critical mentalizing areas in the cerebrum to identify sequences of social actions and then sends signals back to the same cortical mentalizing areas to better prepare for others’ social actions and one’s responses to it.

## Introduction

Although it has an established role in motor functioning and coordination, there is a growing interest in the role of the cerebellum in non-motor mental functions, such as social cognition (Van Overwalle *et al.*, 2014). Recent research has shown that the posterior cerebellum is consistently activated during the process of understanding others’ mental states (e.g. inferring their traits, beliefs and intentions), which is termed ‘Mentalizing’ or ‘Theory of Mind’ (Van Overwalle *et al.*, 2014, 2020a; Guell *et al.*, 2018; Metoki *et al.*, 2021). Also, research showed that posterior cerebellar activity increased with mentalizing complexity such as tracking multiple different mind states (Lewis *et al.*, 2017). Several meta-analyses have reliably identified key areas in the cerebral cortex in the mentalizing processes, including the temporoparietal junction (TPJ), precuneus (PCun), temporal pole (TP) and medial prefrontal cortex (mPFC; Van Overwalle, 2009; Schilbach *et al.*, 2012; Schurz *et al.*, 2014; Molenberghs *et al.*, 2016). However, less is known about how the posterior cerebellum interacts with the cerebral mentalizing network in social cognition.

How does the posterior cerebellum contribute to mentalizing? Inspired by the sequence detection hypothesis of the cerebellum (Leggio and Molinari, 2015), (Van Overwalle *et al.* 2019b) proposed that the posterior cerebellum encodes social sequences of people’s actions, automatizes social sequences that occur frequently and hence immediately detects violations to these acquired sequences. This sequence detection function allows us to understand how social behaviors unfold over time and to react proactively to social actions of others. For example, when a person rummages in his or her pocket and then shows a candy to a child, this sequence of movements signals an invitation for the child to take the candy. It will come as an unwelcome surprise if that person starts eating the candy him- or herself instead. (Van Overwalle *et al.* 2019a) proposed that many social signals and interactions are based on this sort of social action sequences that become routines and so facilitate social understanding and behavior.

In line with this hypothesis, cerebellar patients showed significant difficulties in comprehending the correct sequence of social stories depicted in cartoon-like pictures (picture sequencing task), especially when a good understanding of the story is required to consider divergent beliefs held by other persons (Van Overwalle *et al.*, 2019a). In one of the cartoons, for example, a boy was surprised to see that his chocolates were gone, because he did not know that they were eaten by a girl while he went away playing. To understand how this story unfolds, it is necessary to switch to the boy’s perspective and initial belief that nothing happened in his absence. Using the same picture sequencing task, healthy participants showed stronger activation of the posterior cerebellar Crus I & II for stories involving social beliefs in comparison with nonsocial events (Heleven *et al.*, 2019). The involvement of the posterior Crus I & II has been confirmed in many other tasks that compared generating or remembering sequences of social actions *vs* nonsequencing or nonsocial control conditions (Van Overwalle *et al.*, 2021b), as well as in meta-analyses encompassing social cognition in general (Van Overwalle *et al.*, 2014, 2020a).

It is generally assumed that the sequencing function of the cerebellum is accomplished through multisynaptic communication with the cerebral cortex (Ito, 2008). Early evidence from anatomical studies on animals documented connections between the cerebellum and the cerebral cortex (Glickstein *et al.*, 1985; Schmahmann, 1996; Kelly and Strick, 2003; Suzuki *et al.*, 2012). Subsequent studies on humans using diffusion tensor imaging (DTI) to reconstruct cerebello–cerebral white matter pathways documented many cerebellar interconnections with the cerebral cortex (Sokolov *et al.*, 2014; Karavasilis *et al.*, 2019; Metoki *et al.*, 2021). The specific cerebral areas where these connections make contact may determine the specificity of the cerebellar process (Diedrichsen *et al.*, 2019). In particular, closed-loop circuits, which involve the same cerebellar and cerebral areas as the origin and target of reciprocal connectivity (Kelly and Strick, 2003), may provide the anatomical substrate for the domain-specific interaction. In the social domain, using DTI, Metoki *et al.* (2021) recently documented robust structural connections between cerebellar and cerebral mentalizing areas, including between Crus I and the TPJ, when participants were required to infer intentions of moving shapes. This finding provides initial support for the idea that incoming signals from key cerebral mentalizing areas responsible for intention understanding, such as the TPJ, are forwarded to the cerebellar Crus to identify the sequences of social events and are then fed back to cortical mentalizing areas, producing error signals in case of sequence violations. However, anatomical studies by themselves do not provide evidence for reciprocal functional closed-loop circuits in mentalizing.

The functional connectivity between the cerebellum and the cerebrum in social cognition has been demonstrated using several methodologies. Resting-state parcellation analyses showed that the cerebellum is organized in domain-specific networks and that the posterior cerebellum (especially Crus I & II) and some anterior parts (lobule IX) are topographically mapped to the mentalizing network in the cerebrum (Buckner *et al.*, 2011; Guell *et al.*, 2018). Task-related psychophysiological interaction connectivity analysis supported an intimate cerebello–cerebral mentalizing network by revealing strong functional connections from the posterior cerebellum to cerebral mentalizing areas (Metoki *et al.*, 2021). However, a limitation of these methods is that they are unable to identify the direction of connectivity between the cerebellum and the cerebrum, although this is essential to uncover bidirectional functional closed loops which are key to the sequencing hypothesis (Leggio and Molinari, 2015; Van Overwalle *et al.*, 2019b). Dynamic causal modeling (DCM), in contrast, is a more powerful method that can identify the direction of functional connectivity and that avoids spurious (or indirect) correlations, because it rests on a comprehensive mechanistic model of the causal effects that generate the data. Therefore, this type of ‘bidirectional functional’ connectivity has also been referred to as ‘effective’ connectivity. Earlier DCM analyses have identified bidirectional effective closed loops between the bilateral posterior cerebellum and the bilateral TPJ when people were inferring others’ traits (Van Overwalle *et al.*, 2019c) and comprehending social stories involving other’s beliefs in the picture sequencing task mentioned earlier (Van Overwalle *et al.*, 2020b).

### Implicit social sequence learning

An important feature of the cerebellum is that sequence detection and automatization occur largely implicitly, that is, beyond conscious awareness. This is immediately evident in motor learning such as learning to ride a bike, where verbal instructions aid little in keeping one’s balance. The aim of the current study is to investigate the effective cerebello–cerebral connectivity of mentalizing areas during implicit social sequence learning and compare it with explicit social sequence learning.

**Fig. 1.**
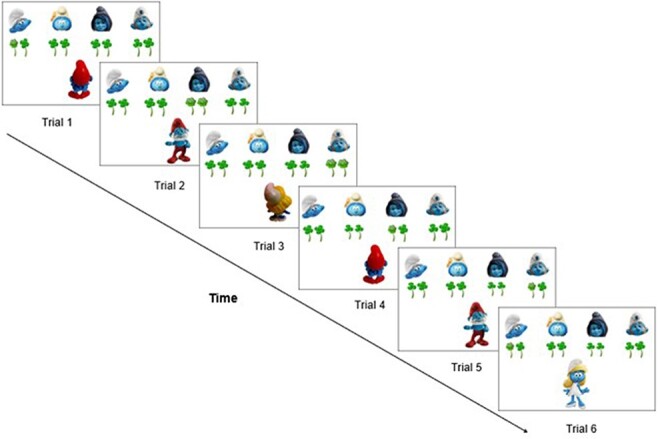
Schematic example showing the first six trials of the standard sequence in the Belief SRT task. In each trial, participants had to report the number of flowers as seen by the protagonists (Papa Smurf or Smurfette). Belief orientations followed a standard sequence. In the Belief SRT task, when the protagonist was oriented to the screen and could see the flowers (true trial), the number of target flowers had to be reported from the current trial; when the protagonist was oriented away from the screen and could not see the flowers (false trial), the number of target flowers had to be reported from the previous true trial from the same protagonist. The number of flowers was random (1 or 2), making the response unpredictable, and dissociating sequence learning from motor responses. Each trial was self-paced, with all stimuli remaining on screen for 3000 ms until a response was given and was followed by a response-stimulus interval of 400 ms before the next trial started (Trials 1 and 2). To illustrate the instructions for the Belief SRT task, in Trial 1, there is one flower that Papa Smurf can see because he is oriented toward the screen, meaning that the correct response is 1. In Trial 2, there are two flowers. Because Papa Smurf is oriented away from the screen, he cannot see the number of flowers on this trial; hence, he still thinks to have received one flower which he last saw on the previous (1st) trial. The correct response is thus again 1.

To measure implicit sequence learning, the classic serial reaction time (SRT) paradigm (Nissen and Bullemer, 1987) was used. In this paradigm, participants rapidly respond to a sequence of stimuli, which are surreptitiously repeated (e.g. spatial locations on a screen). The results show that participants responded faster to the hidden repetition and slower to any sequence disturbances, demonstrating implicit sequence learning. Based on this SRT paradigm, Ma and colleagues (Ma *et al.*, 2021) created a novel Belief SRT task that involves sequences of social beliefs (Figure 1). Participants had to report how many flowers the two protagonists—Papa Smurf and Smurfette—believed they were offered. This task had two critical features. First, crucially for a social belief manipulation, the protagonists were either oriented toward the flowers so that they could see them or oriented away so that they could not see them. When oriented toward the flowers, the protagonist held ‘true’ beliefs about the flowers (i.e. trial 1 in Figure 1) and when oriented away, the protagonist held outdated or ‘false’ beliefs (i.e. trial 2 in Figure 1). Secondly, participants were not informed that there was a repeated sequence of true or false beliefs held by each protagonist (i.e. implicit learning; Ma *et al.*, 2021). Learning this sequence could nonetheless make the task easier, because it allows participants to anticipate each protagonist’s subsequent beliefs and participants could respond accordingly. To test how implicit *vs* explicit instructions contribute to this task, an explicit version of the Belief SRT task was also developed. The explicit task is identical to the implicit learning task except that participants were informed about the existence of a sequence, although the exact sequence was not given (i.e. explicit learning; Ma *et al.*, 2022).

In the implicit and explicit Belief SRT tasks, there was an initial Training phase where the standard sequence was repeated, followed by a subsequent Test phase where the standard sequence was interrupted by random sequences. The initial Training phase captures how the standard sequence is gradually learned during repeated practice, and the later Test phase captures how the learned standard sequence is applied while being occasionally disturbed by random sequences. Of main behavioral interest is the random sequence where a slowing down of responses compared to the standard sequence captures that the repeated sequence was learned and anticipated (Nissen and Bullemer, 1987). Although this behavioral effect was robustly replicated in the implicit and explicit Belief SRT tasks, the typical contrast of the repeated standard sequence against the random sequence (cf. meta-analysis by Janacsek *et al.*, 2020) did not reveal activations of the cerebellum. Therefore, the current study focuses on the Training and Test phases of the standard belief sequence.

Regardless of the implicit or explicit instruction, the Belief SRT task showed that the posterior cerebellar Crus I and lobule VI, and cortical areas like the TPJ and PCun were activated during the initial Training phase. The cerebellar Crus II and (sub)cortical caudate and TP were additionally activated during the later Test phase (Table 1; Ma *et al.*, 2021). Together, these results indicate that distinct areas are involved during different learning phases.

**Table 1.** ROIs for the DCM analysis and total number of participants (*n*) and number of participants at reduced thresholds

	*MNI Coordinate*			Implicit Belief (*n* = 18)		Explicit Belief (*n* = 22)	
Region and anatomical label	*x*	*y*	*z*	<0.1	<1	<0.1	<1
Standard block at Training > standard block at Test							
Cerebellar ROIs							
L Crus I	−40	−70	−40	1		2	2
L Crus II	−25	−75	−40		3	4	4
R Crus II	25	−75	−40	1	6	4	7
L Lob. VI	−24	−54	−20	1		1	1
R Lob. VI	24	−54	−20			2	
Cerebral ROIs							
PCun	0	−60	40				1
L TPJ	−50	−55	25				1
R TPJ	50	−55	25				
Standard block at Test > standard block at Training							
Cerebellar ROIs							
L Crus I	−40	−70	−40		4		2
L Crus II	−25	−75	−40	1	3		1
R Crus II	25	−75	−40		1		
Cerebral ROIs							
L TP	−51	0	−19				
R TP	53	0	−21				
L caudate	−18	6	−4				
L TPJ	−50	−55	25		1		
R TPJ	50	−55	25	1	2		1

Note: L = Left, R = Right, Lob. VI = Cerebellar lobule VI.

It is of interest to note that the posterior cerebellum was slightly more activated during implicit learning compared to explicit learning when participants tried to maintain the learned standard sequence in the later Test phase (Ma *et al.*, 2021).

### Effective connectivity

To analyze the cerebello–cortical effective connections during these implicit and explicit Belief SRT tasks, we applied DCM. As noted before, DCM is a well-established and biologically plausible method that allows us to estimate the strength of the connections from one area to the other and vice versa, that is, in two directions (Friston *et al.*, 2003). This bidirectional connectivity allows us to test the existence of effectively closed loops. DCM estimates fixed and modulatory connections: Fixed (or ‘endogenous’ or ‘intrinsic’) connections reflect the connectivity between brain areas which are unmodulated by various experimental conditions and modulatory connections reflect modulation by conditions, that is, increase or decrease of connectivity depending on condition (Friston *et al.*, 2003). By estimating all (bidirectional) connections at once in a single DCM model, false connectivity caused by indirect connections via other areas is controlled for (Van Overwalle *et al.*, 2019c).

To test the effective cerebello–cerebral connectivity during social sequence learning, we applied DCM to our previous fMRI studies on implicit and explicit Belief SRT tasks (Ma *et al.*, 2021). We hypothesized that there would be effective connectivity, mainly as closed loops, between the posterior cerebellum Crus I & II and the bilateral TPJ as suggested by previous DCM connectivity studies on social reasoning and belief understanding (Van Overwalle *et al.*, 2019c, 2020b). Given the activation in additional brain areas (e.g. cerebellar lobule VI, caudate, PCun and TP) during the Belief SRT tasks mentioned earlier (Ma *et al.*, 2021), we further expected that these brain areas might also be involved and connected. Based on earlier DCM analyses on social cognition where few modulatory connections were found depending on domain (social *vs* nonsocial) or consistency (consistent *vs* inconsistent contexts; Van Overwalle *et al.*, 2019c, 2020b), we expect here also little modularity of the connections depending on the learning phase.

## Method

We applied DCM to prior fMRI studies on implicit and explicit Belief SRT tasks (Ma *et al.*, 2021). We briefly repeat here the main methodological features of these studies and refer to the original articles for more detailed information. After that, we describe the DCM analyses.

### Participants

There were a total of 18 (14 female, mean age 21.2 ± 2.7) healthy, right-handed, Dutch-speaking participants in the implicit Belief SRT task and 22 (17 female, mean age 23.3 ± 4.1) in the explicit Belief SRT task. All of them had normal or corrected-to-normal vision and color perception. To avoid any carry-over effects (Geiger *et al.*, 2018), participants completed the Belief SRT task with either implicit or explicit instruction. All participants gave written informed consent with the approval of the Medical Ethics Committee at the University Hospital of Ghent. Participants were paid 20 euros, and transportation costs were reimbursed in exchange for their participation.

### Stimuli material

In the Belief SRT task (Figure 1A), the target consisted of one or two flowers, appearing in one of four horizontal locations, marked by four little smurfs on the top of the screen. The target flower(s) were presented with clovers as distractors. The two protagonists, Papa Smurf and Smurfette, were each shown individually at the bottom of the screen with their faces oriented to or away from the screen. Participants were told: ‘One of the four little smurfs will give the flowers while Papa Smurf or Smurfette is watching (facing the screen) or not watching (facing you). Papa Smurf and Smurfette count the flowers they receive. Throughout the task you have to track how many flowers Papa Smurf or Smurfette thinks he or she will get (1 or 2). If they are turned with their back to the four smurfs, you have to indicate how many flowers they (remember that they) received the last time’ (Best translation from Dutch).

Participants only received the above information in the implicit task. In the explicit task (Ma *et al.*, 2022), participants received additional information about a sequence they should search for: ‘WATCH OUT! In this task, there is a fixed sequence of Papa Smurf and Smurfette and their orientations (toward or away from the screen). Try to find this sequence as that will make the task easier for you. After the task you will have to demonstrate that you have found this order’ (Best translation from Dutch). Note that participants were explicitly informed about the kind of sequence they should search for (i.e. protagonists and their orientations), avoiding possible misunderstandings such as sequences tied to the flowers’ location (which varied also throughout the experiment in an unrelated sequence). However, participants were not informed about the exact sequence itself so that the task tapped on sequence learning rather than memory processes (Deroost and Coomans, 2018).

### Procedure

Here, we briefly summarize the essential aspects of the sequence learning procedure. For more details, we refer to prior fMRI studies on implicit and explicit Belief SRT tasks (Ma *et al.*, 2021). The whole experimental task consisted of 30 blocks with 32 repeated trials each and was divided into a Training phase (blocks 1–5) and a Test phase (blocks 6 – 30). Participants provided responses with their left middle or index finger (i.e. one or two flowers, respectively) via an MRI-compatible two-button response box.

In an initial Training phase, the standard sequence was repeated throughout five blocks. This standard sequence consisted of 16 trials of protagonists (smurfs) and orientations (beliefs) and was repeated two times per block. In a subsequent Test phase, there were eight standard blocks, identical to those in the Training phase, which were each followed by two types of random blocks: total random block, where protagonist and orientation were totally randomized with the limitation of at most two subsequent trials of the same orientation type, consistent with the standard blocks; random orientation blocks, where the orientation was changed into a different pseudo-random sequence, while the order of protagonists was identical as in the standard blocks (Supplementary Table S1). The last block at the end of the whole task was always a standard block.

### Imaging procedure and preprocessing

Images were collected with a Siemens Magnetom Prisma fit 3T scanner system (Siemens Medical Systems, Erlangen, Germany) using a 64-channel radiofrequency head coil. Stimuli were projected onto a screen at the end of the magnet bore and viewed by way of a mirror mounted on the head coil. Stimulus presentation was controlled by E-Prime 2.0 (www.pstnet.com/eprime; Psychology Software Tools) running under Windows XP. Participants were placed head first and supine in the scanner bore and were instructed not to move their heads to avoid motion artifacts. Foam cushions were placed within the head coil to minimize head movements. First, high-resolution anatomical images were acquired using a T1-weighted 3D MPRAGE sequence (Repetition Time (TR) = 2250 ms, Echo Time (TE) = 4.18 ms, Inversion Time (TI) = 900 ms, Field of View (FOV) = 256 mm, flip angle = 9°, voxel size = 1 × 1 × 1 mm). Secondly, a fieldmap was calculated to correct for inhomogeneities in the magnetic field (Cusack and Papadakis, 2002). Thirdly, whole-brain functional images were collected in a single run using a T2*-weighted gradient multiband echo sequence, sensitive to Blood Oxygenation Level Dependent (BOLD) contrast (TR = 1000 ms, TE = 31.0 ms, FOV = 210 mm, flip angle = 52°, slice thickness = 2.5 mm, distance factor = 0%, voxel size = 2.5 × 2.5 × 2.5 mm, 56 axial slices, acceleration factor GRAPPA = 4).

SPM12 (Wellcome Department of Cognitive Neurology, London, UK) was used to process and analyze the fMRI data. To remove sources of noise and artifact, data were preprocessed. Inhomogeneities in the magnetic field were corrected using the fieldmap (Cusack and Papadakis, 2002). Functional data were corrected for differences in acquisition time between slices for each whole-brain volume, realigned to correct for head movement and co-registered with each participant’s anatomical data. Then, the functional data were transformed into a standard anatomical space (2 mm isotropic voxels) based on the ICBM152 brain template (Montreal Neurological Institute). Normalized data were then spatially smoothed (6 mm full width at half maximum) using a Gaussian Kernel. Finally, using the Artifact Detection Tool (ART; http://web.mit.edu/swg/art/art.pdf; http://www.nitrc.org/projects/artifact_detect), the preprocessed data were examined for excessive motion artifacts and for correlations between motion and experimental design, and between global mean signal and experimental design. Outliers were identified in the temporal differences series by assessing between-scan differences (*Z*-threshold: 3.0 mm, scan to scan movement threshold: 0.5 mm; rotation threshold: 0.02 radians). These outliers were omitted from the analysis by including a single regressor for each outlier. A default high-pass filter was used for 128 s, and serial correlations were accounted for by the default auto-regressive (1) model.

### Statistical analysis of neuroimaging data

The statistical analyses were performed using the general linear model of SPM12 (Wellcome Department of Cognitive Neurology, London, UK). At the first (single participant) level, an event-related design for measuring transient activity across trials was modeled by entering separate regressors for the trials of interest: two regressors for the trials in the standard blocks at the Training and Test phases (i.e. standard block at Training and standard block at Test), two regressors for the trials in the total random blocks and the trials in the random orientation blocks at the Test phase (i.e. total random at Test and random orientation at Test) and two additional regressors of no interest for pauses and error trials. This last regressor involved incorrect trials as well as one trial after each incorrect trial, because these latter trials may be affected by error processing on the prior trial.

At the second (group) level, we conducted a within-participant one-way analysis of variance and defined *t*-contrasts between regressors of interests. As mentioned earlier, the often-used fMRI contrast of the standard sequence at Test > Random sequence at Test (cf. meta-analysis by Janacsek *et al.*, 2020) did not reveal any activation of the cerebellum in the original fMRI studies (Ma *et al.*, 2021). Therefore, we focus the analysis here on two contrasts related to different phases in learning the standard belief sequence.

1. Initial training: brain activations during initial learning of the standard sequence are tested by the contrast: standard block at Training > standard block at Test.
2. Later test: brain activations during late learning in a context of sequence violations (in the Test phase) are tested by the contrast standard block at Test > standard block at Training. Note that this contrast does not show the mere late phase of sequence learning, as it also involves reinstating the learned standard sequence after random sequences.

### Dynamic causal modeling

#### Selection of regions of interest

Since we hypothesized that key mentalizing areas in the posterior cerebellar Crus I & II and in the cortical TPJ would be involved during SRT learning, we took a priori regions of interest (ROIs) of these areas from previous meta-analyses (Table 1; Van Overwalle and Baetens, 2009; Van Overwalle *et al.*, 2020a), which were also used in previous DCM studies (Van Overwalle *et al.*, 2019c, 2020b).

There were additional activations of cerebellar (e.g. lobule VI) and cerebral areas (e.g. PCun and TP) related to sequence learning and mentalizing processes in the implicit and explicit Belief SRT tasks. However, instead of using the peak coordinates from these studies, we took a more generalized approach by taking a priori coordinates from meta-analyses (Hardwick *et al.*, 2013; Schurz *et al.*, 2014; Van Overwalle and Baetens, 2009; Table 1). To verify that these meta-analytic coordinates would also fit the activation data from the Belief SRT tasks, we constructed spheres around their centers with radius = 15 or 10 mm for the cerebrum and cerebellum/subcortical areas, respectively (given the smaller size of the cerebellum and subcortical areas). A small volume correction analysis revealed significant activations in the bilateral cerebellar lobule VI and PCun during the initial Training phase (i.e. standard block at Training > standard block at Test) and additional clusters in the bilateral TP and caudate during the later Test phase (i.e. standard block at Test > standard block at Training). Thresholds were similar as in the original Belief SRT studies, that is, cluster-forming threshold of *P* < 0.001 (uncorrected) with a minimum extent of 10 voxels and a cluster-wise significance level of *P* < 0.05 with family-wise error correction for multiple comparisons.

Individually tailored cerebral ROIs were created by extracting the time series using the eigenvariate within a sphere with a radius of 8 or 5 mm (for the cerebrum and cerebellum, respectively) around the nearest local maximum within the corresponding ROIs listed above, using a whole-brain threshold of the contrast at *P* < 0.05 (uncorrected). All voxels contributing to the ROI were conducted on an *F*-contrast involving the comparison of interest and adjusted using an *F*-contrast involving all experimental effects. If the individual ROI did not contain a peak surviving the *P *< 0.05 uncorrected threshold, the same procedure was repeated with reduced thresholds *P* < 0.10 and *P *< 1.00 (uncorrected) so that the time series of all ROIs were included for all participants (see Table 1 for details). In the latter case of *P* < 1.00, ROIs were centered around the group-based centers (Zhou *et al.*, 2007). This procedure was used because pairwise exclusion is not possible in a DCM analysis as all time series from all ROIs are required. Setting a more tolerant threshold in some individual cases implies an optimal compromise between maximizing the effect of interest at the individual level while having all participants in the DCM analysis so that the results are not biased by excluding some participants (Zhou *et al.*, 2007).

#### DCM specification.

We used exactly the same procedure as in the earlier DCM analysis by (Van Overwalle *et al.* 2020b) which followed the steps described in Friston *et al.* (2016) and Friston *et al.* (2015) and detailed in https://en.wikibooks.org/wiki/User:Peterz/sandbox.

First, a full DCM was specified and estimated for each participant using SPM12 (cf. SPM procedure: spm_dcm_fit). A full model allows all connectivity parameters in all directions to be freely estimated. We specified a bilinear deterministic DCM without centering around the mean (Friston *et al.*, 2003), which included (i) all forward and backward fixed connections between the ROIs, (ii) all the modulatory connections or parameters that reflect condition changes due to each experimental condition and (iii) direct input parameters that reflect the input driving the activity in the ROIs in all experimental and control conditions. Stated differently, the driving input in Matrix C consists of one vector with all the onsets of all experimental and control conditions combined as one input, and the modularity connections in Matrix B are specified only for the experimental conditions so that both matrix inputs are nonredundant to each other (Hillebrandt *et al.*, 2014).

Secondly, we constructed a parametric empirical Bayes (PEB) model for the whole group of participants over all parameters (cf. SPM procedure: spm_dcm_peb). This makes it possible to estimate the effective connectivity averaged across all participants (cf. group average), considering the within-participants variability on the connectivity parameters, unlike a classical test (e.g. *t*-test) which ignores the estimated uncertainty (variance) about the connection strengths. Moreover, a group-level PEB allows us to control for differences between sets of studies by treating them as covariates, for example, any differences in the behavioral measures and procedures (Friston *et al.*, 2016).

Thirdly, we automatically pruned away any connectivity parameter from the group-level PEB which did not contribute to the model evidence using Bayesian model reduction (cf. SPM procedure: spm_dcm_peb_bmc). This approach has the advantage that any reduced model at the group level can be estimated efficiently without having to re-estimate the reduced models at the lower level (single-participant levels) and is therefore recommended (Friston *et al.*, 2015). Specifically, a greedy search iteratively prunes connection parameters from the full model until model evidence starts to decrease so that the most relevant nested models from the full PEB model are tested (a greedy search is recommended because the model space of all possible nested models is too large to be fully evaluated). Bayesian model averaging of the parameters of the best 256 pruned models is applied and used for group inferences (Zhou *et al.*, 2018, p. 707, 2.7.4) and so determines the winning model empirically. We considered connectivity parameters as significant given a posterior probability of *P* > 0.95 (based on model comparisons with and without each parameter). This Bayesian approach both for first-level connectivity analysis (DCM) as well as group-level inference (PEB) on connectivity parameters eschews the multiple comparisons problem (Friston *et al.*, 2003, p. 1276, 1.3). We applied these recent developments in DCM analysis to ensure that the connections revealed by the analyses are entirely data-based.

Moreover, to rule out multicollinearity in the DCM estimates due to the relatively large number of ROIs within the smaller volume of the cerebellum, we ran two additional reduced models for the two Belief SRT tasks. One DCM model included only the hypothesized mentalizing cerebellar and cerebral areas (i.e. the left Crus I, the bilateral Crus II and the bilateral TPJ). Another DCM model included only robustly significant ROIs. These results did not change the main results appreciably and are therefore reported in Supplementary Tables S2–S5.

## Results

The fixed and modulatory estimates of the connectivity between the cerebral and cerebellar ROIs of the reduced model (after pruning) in the implicit and explicit Belief SRT tasks are listed in Table 2 for the initial Training phase and in Table 3 for the later Test phase. We first report the fixed estimates which reflect the intrinsic connections between regions, and then we report the modulatory estimates which reflect the modulation of connections depending on the learning phases. We do not report the direct inputs, because none of the driving inputs reached significance.

**Table 2.** Averaged connections in units of 1/s (Hz) for the contrast: standard block at Training > standard block at Test

From to	L Crus I	L Crus II	R Crus II	L Lob. VI	R Lob. VI	PCun	L TPJ	R TPJ
Implicit Belief SRT task								
	Fixed connectivity							
L Crus I	*−0.67* **	0.05*	−0.08**	0.03*		−0.06**	0.14**	0.13**
L Crus II		*−0.67* **					0.12**	
R Crus II	−0.12**	0.33**	*−0.64* **	0.10**	−0.13**			
L Lob. VI		−0.09**	0.04*	*−0.79* **	0.29**		0.19**	−0.28**
R Lob. VI	−0.14**			0.13**	*−0.71* **	−0.16**		0.23**
PCun	−0.11**			0.10**	0.11**	*−0.53* **		0.14**
L TPJ	0.09**		0.04*				*−0.65* **	
R TPJ	0.23**		0.11**	0.07**	−0.11**	0.08**	−0.18**	*−0.54* **
	Modulatory connectivity							
L Crus I	*−0.03*	0.02		−0.01	0.01	0.02	−0.02	0.01
L Crus II	−0.01	*−0.30* *	−0.01	0.01	0.01	0.01	−0.03	−0.02
R Crus II	−0.02	−0.01	*−0.41* *	0.02	0.04		−0.03	−0.01
L Lob. VI	−0.02	0.02	0.04	*−0.32* *	−0.34**	0.03	−0.02	0.02
R Lob. VI	0.03	0.01	0.01	0.02		0.03	0.04	−0.05
PCun	0.02	0.01	−0.02	−0.01	0.04	*−0.02*	0.02	0.01
L TPJ	−0.04	−0.04	−0.02	0.01	0.02	0.01	*−0.32* *	0.01
R TPJ	−0.05	−0.04	−0.05	0.01	0.02	−0.04	−0.01	*−0.62* **
Explicit Belief SRT task								
	Fixed connectivity							
L Crus I	*−0.65* **	0.18**		−0.12**	0.19**	0.04*	0.08**	−0.18**
L Crus II	0.13**	*−0.44* **	−0.06*			0.16**		−0.05*
R Crus II	0.14**		*−0.47* **		−0.05*	0.12**		
L Lob. VI				*−0.47* **		0.20**	−0.09**	0.10**
R Lob. VI	0.13**		−0.17**		*−0.28* **	0.36**		
PCun	−0.11**	0.22**		−0.11**		*−0.72* **	−0.06*	0.24**
L TPJ				0.10**			*−0.74* **	−0.08**
R TPJ	−0.05*			−0.06**		0.29**	−0.07**	*−0.35* **
	Modulatory connectivity							
L Crus I	*−0.03*	−0.02	0.03	0.06		−0.05	0.05	0.02
L Crus II	−0.01	*−0.03*	0.02	0.07	0.04	−0.02	−0.02	
R Crus II	−0.01		*−0.03*	0.06	0.03	−0.02	−0.03	
L Lob. VI	−0.03	−0.01	−0.01	*−0.54* **	−0.03	−0.03	−0.05	−0.01
R Lob. VI	−0.05	−0.02	0.01		*−0.50* **	−0.06	−0.04	0.01
PCun	−0.01	−0.01		0.01	0.01	*−0.30* *	−0.24*	0.03
L TPJ	−0.05	0.03	0.02	−0.03	0.04	0.01	*−0.02*	0.07
R TPJ		0.04	−0.01	0.04	−0.02	−0.07	−0.02	*−0.72* **

Notes: L = left, R = right, Lob. VI = cerebellar lobule VI. Cell entries refer to the connections from the top row ROIs to the left column ROIs with posterior probability. Empty cells denote connectivity = 0. Bold denotes the closed loops. *Italic* denotes the self-inhibitions in diagonal cells. Light gray denotes the cerebellar–cortical connectivity.

** *P* > 0.95,

* *P* > 0.50.

**Table 3.** Averaged connections in units of 1/s (Hz) for the contrast: standard block at Test > standard block at Training

From to	L Crus I	L Crus II	R Crus II	L TP	R TP	L Caudate	L TPJ	R TPJ
Implicit Belief SRT task								
	Fixed connectivity							
L Crus I	*−0.71* **		−0.11**	−0.10**		0.10**	0.13**	
L Crus II		*−0.40* **		0.09**		−0.11**	0.10**	0.21**
R Crus II	−0.10**		*−0.61* **	−0.17**			0.19**	0.16**
L TP	0.05*	0.10**	−0.22**	*−0.51* **	0.09**		0.08**	0.10**
R TP		−0.04*			*−0.51* **	−0.08**	0.13**	0.09**
L caudate		−0.11**			−0.22**	*−0.79* **	0.17**	0.10**
L TPJ	−0.09**	0.18**	−0.23**	0.05*	−0.08**	0.13**	*−0.71* **	0.14**
R TPJ		0.05*	−0.10**				0.12**	*−0.68* *
	Modulatory connectivity							
L Crus I	*−0.02*	−0.01	0.04	−0.28*	0.03	0.01		0.03
L Crus II	0.02	*−0.02*	−0.02	−0.04	−0.01	0.02		−0.01
R Crus II	0.03	−0.05	*0.01*	0.02	−0.03			0.02
L TP	−0.03	−0.01	−0.02	*−0.03*	−0.04	0.01	−0.04	0.02
R TP	0.03	−0.02	−0.05	−0.01	*−0.02*	0.03	−0.04	−0.01
L caudate	−0.02	0.02	−0.04	−0.01	0.03	*−0.37* *	−0.05	0.01
L TPJ	0.01	−0.03	0.00	−0.03	−0.02	−0.03	*−0.23* *	0.01
R TPJ		−0.05	0.03		−0.01	−0.01	−0.05	*−0.30* *
Explicit Belief SRT task								
	Fixed connectivity							
L Crus I	*−0.43* **	0.12**			0.09**		0.04*	−0.04*
L Crus II	0.11**	*−0.25* **	0.16**	−0.10**	−0.19**	0.14**		0.21**
R Crus II	−0.14**	0.16**	*−0.41* **			0.09**	0.18**	
L TP	−0.13**	−0.13**	0.20**	*−0.25* **	0.11**			0.23**
R TP		−0.13**			*−0.35* **	0.14**	0.20**	
L caudate		−0.11**	0.05*	−0.17**		*−0.60* **	0.10**	0.20**
L TPJ		−0.22**		−0.10**	0.08*		*−0.48* **	0.43**
R TPJ	−0.17**						0.26**	*−0.48* **
	Modulatory connectivity							
L Crus I	*−0.02*	−0.04	0.01	−0.04	−0.01		0.01	0.03
L Crus II	−0.02	*−0.24* *	−0.18*		0.03	−0.03	−0.02	−0.01
R Crus II	0.03	−0.01	*−0.03*	−0.04	−0.03	−0.02	−0.06	−0.03
L TP	−0.05	−0.02	−0.05	*−0.01*	−0.06		−0.02	−0.05
R TP	−0.02	−0.01	−0.03	−0.02	*−0.25* *	−0.06	−0.20*	−0.02
L caudate	−0.03	−0.04	−0.01	0.01		*−0.61* **	−0.06	−0.05
L TPJ	−0.04	0.01	0.01	−0.04	0.01	0.06	*−0.03*	−0.05
R TPJ	−0.02	−0.03	−0.01	0.00	0.03	0.03	−0.03	*−0.02*

Notes: L = left, R = right. Cell entries refer to the connections from the top row ROIs to the left column ROIs with posterior probability. Empty cells denote connectivity = 0. Bold denotes the closed loops. *Italic* denotes the self-inhibitions in diagonal cells. Light gray denotes the cerebellar–cortical connectivity.

** *P* > 0.95,

* *P* > 0.50.

Of most interest are the estimates in the off-diagonal cells of Tables 2 and 3. These values reflect how much activation in the source ROI (i.e. the top row) changes the activation in the target ROI (i.e. the left column), which is expressed in units per second or 1/s (Hz). Here, positive estimates indicate excitation, and negative estimates indicate inhibition between ROIs. Of less interest are the estimates in the diagonal cells of Tables 2 and 3 which correspond to self-connections, with positive estimates indicating greater self-inhibition, whereas negative estimates indicate less self-inhibition than the default (−0.5 Hz). Decreased self-inhibition suggests increased sensitivity to input from other brain areas (Zeidman *et al.*, 2019).

As hypothesized, we found fixed bidirectional connectivity (i.e. closed loops) between the posterior cerebellar Crus I & II and the bilateral TPJ in both implicit and explicit Belief SRT tasks during both learning phases. Also, we found that the posterior cerebellum was linked to other hypothesized cerebral areas (i.e. PCun, TP and caudate). Additionally, we did not find many modulations. This is supported by the following detailed results.

### Connectivity in the initial training of the standard sequence

We first contrasted the standard blocks in the initial Training phase *vs* the standard blocks in the later Test phase to find out how the ROIs interact with each other during the initial learning of the belief sequence. For each task, we first discuss the ‘fixed’ connectivity between the cerebellum and the cerebrum, then within the cerebrum and then within the cerebellum. This is followed by a short discussion of modular connectivity.

**Fig. 2.**
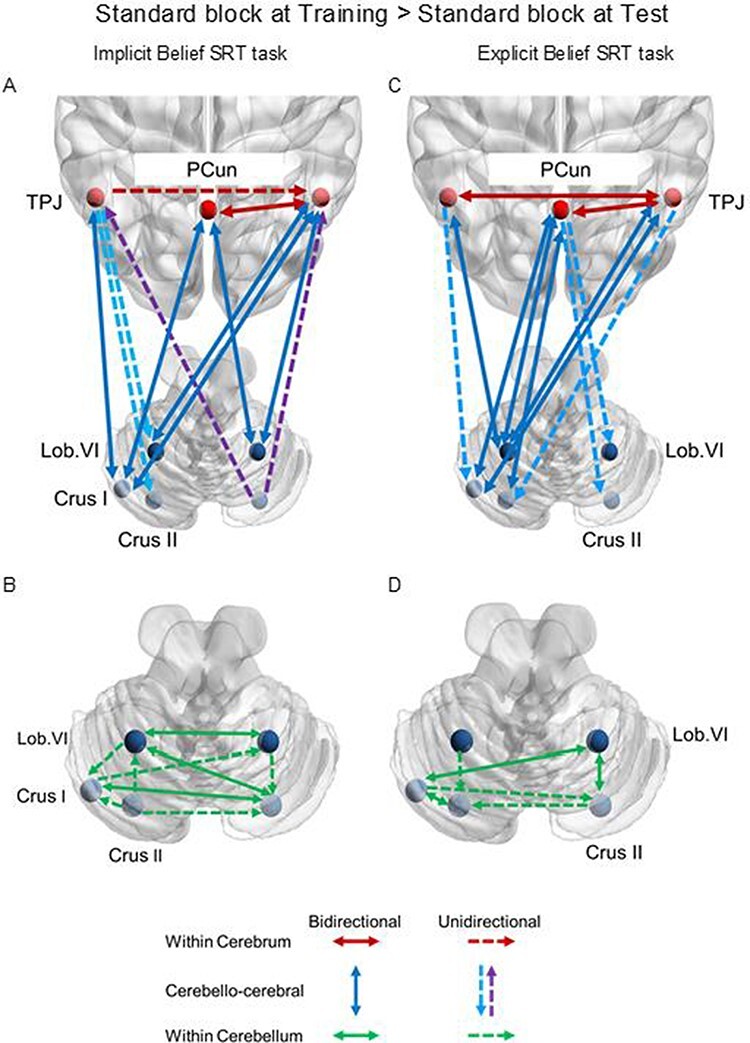
Fixed connections retained in the reduced model for the contrast: standard block at Training > standard block at Test, including cerebellar Crus and lobule VI, TPJ, PCun for the implicit Belief SRT task (A, B) and for the explicit Belief SRT task (C, D). (A–D) Solid arrows indicate the bidirectional connections; dashed arrows indicate the unidirectional connections. (A, C) Dark blue arrows involve the bidirectional connections between the cerebrum and the cerebrum, light blue arrows involve the top-down connections from the cerebrum to the cerebellum, purple arrows involve the bottom-up connections from the cerebellum to the cerebrum. Red arrows involve the connections within the cerebrum. (B, D) Green arrows involve the connections within the cerebellum. The left and right areas represent the left and right of the figure.

For the implicit Belief SRT task (Table 2; Figure 2A), we found significantly closed loops between left Crus I, bilateral lobule VI and the right TPJ and a closed loop between left Crus I and the left TPJ, but only unidirectional links from the right Crus II to the bilateral TPJ. Closed loops were also found between the PCun and left Crus I and the right lobule VI. Within the cerebrum, the PCun was bidirectionally connected with the right TPJ, and the left TPJ was unidirectionally linked to its right counterpart. As shown in Figure 2B, within the cerebellum, most cerebellar ROIs were connected via closed loops to each other, except for a limited number of unidirectional links: on the left hemisphere from Crus II to Crus I, from the Crus II to lobule VI and from the lobule VI to the Crus I; on the right hemisphere, from lobule VI to Crus II, between hemispheres, from the left Crus I to the right lobule VI and from the left Crus II to the right Crus II.

The modularity connections showed decreased self-inhibition in the bilateral Crus II, left lobule VI and bilateral TPJ during the initial Training phase. There were almost no modulations of the connections between ROIs, except for a weaker connection from the left to the right lobule VI during the initial Training phase.

We observed a largely similar pattern of connectivity for the explicit Belief SRT task (Table 2; Figure 2C, D) with again a few unidirectional exceptions which are, however, different than during the implicit task. As can be seen in Figure 2C, we found again significantly closed loops between the cerebral ROIs and the bilateral TPJ, except for unidirectional links from the left TPJ to the left Crus I and from the right TPJ to the left Crus II. Significant connections were also found between the PCun and cerebellar ROIs, and they were more numerous here: closed loops to all left cerebellar ROIs and unidirectional links to all the right ROIs. Within the cerebrum, we see again the same links between the left and right TPJ and between the PCun and the right TPJ, but they were now all closed loops. As shown in Figure 2D, within the cerebellum, some cerebellar ROIs were connected via closed loops to each other: on the left hemisphere between the Crus I and Crus II, on the right hemisphere between lobule VI and Crus II and between hemispheres between left Crus I and right lobule VI. Unidirectional links were also observed on the left hemisphere from lobule VI to Crus II, between hemispheres from the left Crus I to the firth Crus II and from the right Crus II to the left Crus II.

The modularity connections showed significantly decreased self-inhibitions in the bilateral lobule VI, PCun and right TPJ during the initial Training phase. There were again almost no modulations of the connections, except for a weaker connection from the left TPJ to the PCun given the initial Training phase.

### Connectivity in the later test of the standard sequence

We next used the reverse contrast of the later Test phase *vs* the initial Training phase to reveal the connectivity during maintaining the learned belief sequence in the later Test phase. We use the same order as before. For each task, we first discuss the ‘fixed’ connectivity between the cerebellum and the cerebrum, then within the cerebrum and then within the cerebellum, followed by a brief discussion of modular connectivity.

**Fig. 3.**
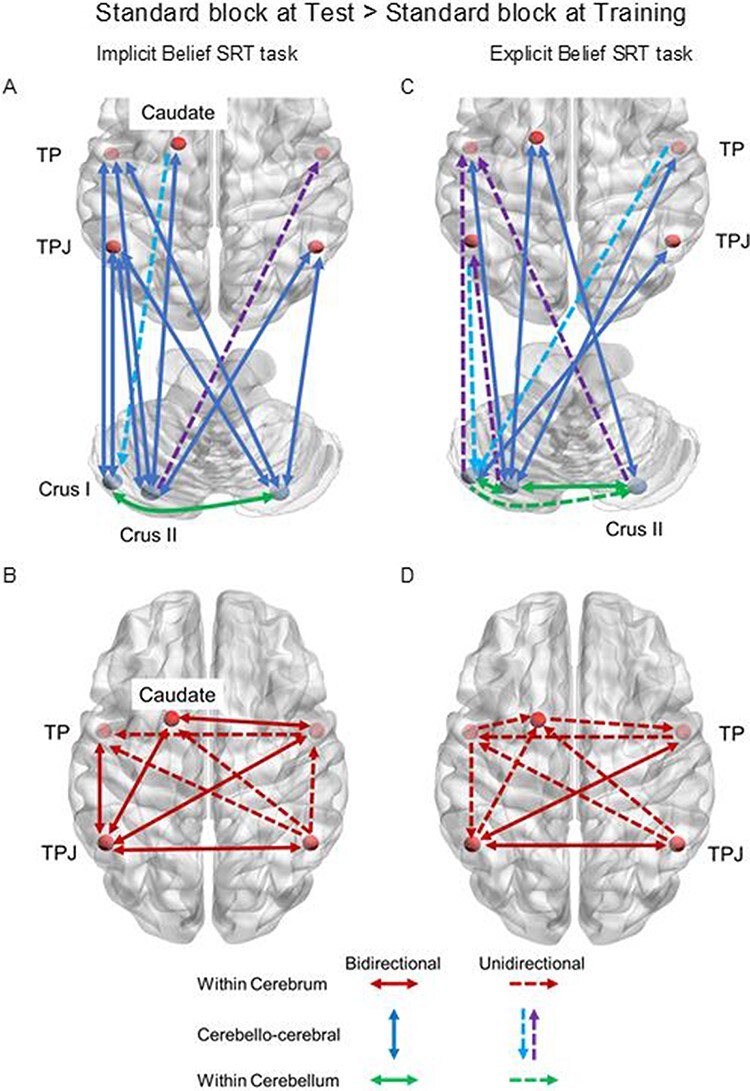
Fixed connections retained in the reduced model for the contrast: standard block at Test > standard block at Training, including cerebellar Crus, TPJ, TP and caudate, for the implicit Belief SRT task (A, B) and explicit Belief SRT task (C, D). (A–D) Solid arrows indicate the bidirectional connections; dashed arrows indicate the unidirectional connections. (A, C) Dark blue arrows involve the bidirectional connections between the cerebrum and the cerebrum, light blue arrows involve the top-down connections from the cerebrum to the cerebellum, purple arrows involve the bottom-up connections from the cerebellum to the cerebrum. Green arrows involve the connections within the cerebellum. (B, D) Red arrows involve the connections within the cerebrum. The left and right areas represent the left and right of the figure.

For the implicit Belief SRT task (Table 3; Figure 3A), we found again significantly closed loops between the cerebral ROIs and the bilateral TPJ. The left Crus I was connected via a closed loop to the left TP and received a link from the left caudate. There were significantly closed loops between the bilateral Crus II and additional (sub)cortical areas (i.e. bilateral TP and left caudate), except for a unidirectional link from the left Crus II to the right TP. Within the cerebrum (Figure 3B), most cerebral ROIs showed closed loops with each other, except for a limited number of unidirectional links: on the right hemisphere from the TPJ to TP, between hemispheres from the right TP to its left counterpart and from the right TPJ to the left TP and to the left caudate. Within the cerebellum, the left Crus I showed a closed loop with the right Crus II.

The modularity connections showed significantly decreased self-inhibitions in the left caudate and the bilateral TPJ but no change of self-inhibitions in the cerebellum. There was a decreased connection from the left TP to the left Crus I given the later Test phase.

For the explicit Belief SRT task, we see a similar pattern of connectivity as the implicit counterpart but now with more unidirectional than closed loops (Table 3; Figure 3C, D). As can be seen in Figure 3C, most closed loops between the cerebral ROIs and bilateral TPJ dropped, except for one closed loop between the left Crus I and the right TPJ. Instead, there were numerous unidirectional connections: from the left Crus II to the left TPJ, from the left TPJ to the left Crus I and from the bilateral TPJ to the contralateral Crus II. Again, the left Crus I showed a unidirectional link to the left TP and received a link from the right TP. We also found closed loops between the bilateral Crus II and the additional (sub)cortical areas (i.e. left caudate and bilateral TP), except for a unidirectional link from the right Crus II to the left TP. Within the cerebrum, some cerebral ROIs were connected via closed loops to each other: between the bilateral TPJ, between the left TPJ and the right TP. Unidirectional links were also observed on the left hemisphere from the TP to the TPJ and from the TPJ to the caudate; between hemispheres from the right TP to the left TPJ, from the right TPJ to the left TP and from the left caudate to the right TP. Within the cerebellum, most cerebellar ROIs were connected via closed loops to each other, except for a unidirectional link from the left Crus I to the right Crus II.

The modularity connections showed significantly decreased self-inhibitions in the left Crus II, right TP and left caudate. There were decreased connections from the right to the left Crus II and from the left TPJ to the right TP given the later Test phase.

### Summary

To sum up, in line with our hypothesis, many connections revealed closed loops between the posterior cerebellum and the bilateral TPJ during both implicit and explicit Belief SRT tasks. For both tasks, additional cerebellar ROIs were also linked to cerebral mentalizing areas. There were numerous (uni- and bidirectional) connections between the cerebellar lobule VI and the bilateral TPJ and the PCun during the initial Training phase (in comparison with the Test phase) and between the posterior cerebellum and the bilateral TP and left caudate during the later Test phase (in comparison with the Training phase). Additionally, there were numerous (uni- and bidirectional) connections within the cerebellum and the cerebrum. Another important observation is that the connectivity patterns were largely similar between the implicit and explicit tasks, although there were more closed loops in the implicit tasks. All connections showed positive as well as negative estimates without a clear downward *vs* upward pattern. Modulations of cerebellar and cerebral self-inhibitions were rare, and modulations of connections between ROIs were largely absent.

## Discussion

In the current study, we used DCM to investigate dynamic cerebello–cerebral connectivity, particularly within the posterior cerebellum and the bilateral TPJ, during implicit and explicit Belief SRT tasks (Ma *et al.*, 2021). After pruning away connections that did not significantly contribute to the model, the DCM analysis offered a model of effective connectivity that provided the best fit with the group-level data of the implicit and explicit tasks. Consistent with our hypothesis, we found that the posterior cerebellum Crus I & II and the bilateral TPJ were connected via closed loops. Moreover, we found that additional brain areas (the cerebellar lobule VI, PCun, TP and caudate) were bi- or unidirectionally linked to the posterior cerebellum and TPJ and so further contributed to belief sequence learning.

Overall, our current study supports the idea that the posterior cerebellum synchronizes neural activation with related mentalizing cerebral areas (i.e. TPJ, PCun and TP) during learning and maintaining a given belief sequence. It is important to emphasize that our ROIs for the DCM analysis were based on earlier meta-analyses and that we used a data-driven greedy search algorithm to reveal significant connections. In this way, a potential bias in favor of our hypothesis was strongly reduced.

The Belief SRT task is the first one to investigate implicit sequence learning in the social mentalizing domain and reveals distinct cerebellar processes and topography compared to nonsocial domains. This is attested by the observation that earlier research on visuomotor sequencing tasks revealed activation in more anterior cerebellar parts (Hardwick *et al.*, 2013; Janacsek *et al.*, 2020), whereas the Belief SRT task robustly activated the posterior cerebellum (Ma *et al.*, 2021). Despite this distinct topography, the current DCM analysis demonstrates that cerebellar and cerebral mentalizing areas are functionally connected and continuously exchange neural signals.

### Many cerebello–cerebral connections have closed-loop properties

In our DCM analysis, the so-called ‘fixed’ cerebello–cerebral connections reflected systematic links among brain areas independent of the initial Training and later Testing phases. In line with our hypothesis, for both implicit and explicit Belief SRT tasks, we found fixed connectivity, mainly as effectively closed loops, between the posterior cerebellum Crus I & II and the bilateral TPJ. The TPJ considered to be a key area for shifting to others’ perspectives and inferring their transient mental states (Van Overwalle, 2009; Schurz *et al.*, 2013). The current analysis confirms earlier DCM studies documenting closed-loop connectivity between the posterior cerebellum Crus II and the TPJ during social mentalizing (Van Overwalle *et al.*, 2019c, 2020b), and our findings extend this functional synchronization to the left posterior cerebellum Crus I.

The current analysis also revealed additional fixed cerebello–cerebral effective closed loops between the posterior cerebellum and other neocortical mentalizing areas, including the PCun and TP. The PCun contributes to the mentalizing process by constructing mental images and social contexts related to different perspectives (Frith and Frith, 2006; Van Overwalle and Baetens, 2009; Schurz *et al.*, 2013). The TP is believed to store social knowledge about others, such as social rules and etiquette (Schurz *et al.*, 2013; Molenberghs *et al.*, 2016). Understanding the different beliefs that protagonists maintain in a variety of situations is an indispensable process supported by these two areas so that their synchrony with cerebellar areas likely contributed to performance in the Belief SRT tasks. These findings extend earlier DCM studies on social mentalizing which found only unidirectional connections from the right cerebellar Crus II to the PCun (Van Overwalle *et al.*, 2019c, 2020b). Overall, the closed loops identified in this analysis support the assumption put forward by (Van Overwalle *et al.* 2019b) that incoming social signals from mentalizing areas in the neocortex are sent further to the posterior cerebellum to identify and automatize their sequences and are then are fed back to cerebral mentalizing areas to affirm ongoing social behaviors or to warn and prepare for unexpected social violations.

The effectively closed loops observed here are supported by anatomical research documenting white matter connections that form closed loops between cerebellar and cerebral cortices. Using DTI on healthy adults, Karavasilis *et al.* (2019) found that the cerebellum is anatomically connected with the frontal and temporal cortices and to a lesser extent to the parietal cortex. A more recent DTI study by Metoki *et al.* (2021) provided evidence for white matter connections between mentalizing areas in the cerebellum (i.e. the posterior Crus I & II) and the cerebral cortex (i.e. mPFC, PCun, TP and TPJ). Interestingly, in that study, the right TPJ revealed strong contralateral pathways toward the cerebellum, while in the reverse direction, the cerebellum displayed weaker contralateral pathways to the TPJ and stronger ones to the mPFC, PCun and TP.

### Other subcortical areas contribute to connectivity with the mentalizing neocortex

This analysis also found that the bilateral cerebellar lobule VI and the left caudate were linked to cerebral and cerebellar mentalizing areas via numerous closed loops. A recent study on social intentions inferred from moving geometrical shapes also revealed strong connectivity between the cerebellar lobule VI and mentalizing areas in the cerebral cortex (Metoki *et al.*, 2021). The cerebellar lobule VI and the caudate were consistently found in recent meta-analyses on visuomotor learning in classical SRT tasks (Hardwick *et al.*, 2013; Janacsek *et al.*, 2020). Recent DCM analyses on visuomotor sequence learning revealed connections between the cerebellum and putamen (including caudate (Tzvi *et al.*, 2015; Liebrand *et al.*, 2020). Because these subcortical areas have never been implicated in other DCM analyses related to social mentalizing as far as we are aware (Van Overwalle *et al.*, 2019c, 2020b), this may suggest that the cerebellar lobule VI and the caudate subserve sequence learning not only in visuomotor contexts but also in some social settings. Alternatively, this may suggest that these areas are specific to some key aspects of the SRT paradigm (i.e. rapidly responding to sequential stimuli with minimal motor actions; Hardwick *et al.*, 2013), rather than general visuomotor or social sequence learning processes.

### Implicit sequence learning reveals more closed loops than explicit sequence learning

We observed similar patterns of connectivity between the implicit and explicit Belief SRT tasks. This implies that both types of belief sequence learning, in a social context, share the same effective connectivity within and between the cerebellar and cerebral mentalizing areas. However, interestingly, there were more within-cerebellar and cerebello–cerebral closed loops in implicit belief sequence learning compared to explicit learning. This seems consistent with the general assumption that the cerebellum is specifically involved in implicit learning and automatization (Taylor *et al.*, 2010; Morgan *et al.*, 2021). For instance, cerebellar patients had impaired performance when implicitly learning a sequence of a shape’s locations but had comparable performance when they were explicitly told to learn and reproduce a given letter sequence (Morgan *et al.*, 2021). It will be of interest for future studies to test behavioral performance and effective cerebello–cerebral connectivity for patients with cerebellar deficits during the completion of the implicit and explicit Belief SRT task. This will allow us to gain insight into how the cerebellum might go awry in social thinking and tailor diagnostic instruments for mentalizing sequencing to the specific needs of these clinical populations.

### Little modulation of cerebello–cerebral connectivity

We did not find many modulations of cerebello–cerebral connectivity induced by the initial Training phase or the later Test phase. For both the implicit and explicit versions, the results revealed a few suppressive modulations within the cerebellum or within the cerebrum, and almost none between the cerebellum and cerebrum, when comparing the initial Training with the Test phase or vice versa. These limited modulations are consistent with earlier DCM research on explicit inferences of social beliefs and traits (Van Overwalle *et al.*, 2019c, 2020b). Note, however, that the Belief SRT tasks measured implicit (and explicit) learning of belief-related sequences, but not of implicit mentalizing itself (i.e. participants were explicitly told to mentalize protagonists’ beliefs). Overall, this seems to indicate that neural synchrony between the cerebello–cerebral mentalizing areas is rather robust and largely unaffected by task changes, such as learning phase, degree of implicitness or type of social inference (e.g. trait or belief). This observation needs to be followed up in future research.

### No clear pattern of positive and negative connections

Although the present analysis largely confirms the hypothesized cerebello–cerebral connectivity between mentalizing areas, there was no clear pattern of positive and negative values for these connections. In earlier DCM analyses on social mentalizing, researchers found that all top-down connections from the TPJ to the posterior cerebellum were positive, and all bottom-up connections were negative (Van Overwalle *et al.*, 2019c, 2020b). This pattern was absent in the current study, where downward and upward estimates between the bilateral TPJ and the posterior cerebellum showed mixed signs of estimates. We ran additional DCM analyses with reduced sets of ROIs to rule out that this was caused by the large number of ROIs within the smaller volume of the cerebellum, which may have led to a statistical problem of multicollinearity and hence a contradictory pattern of estimates. These reduced analyses, however, showed largely the same valence of connectivity as the original analyses. Another methodological explanation is that the earlier DCM analyses by Van Overwalle and colleagues used quite large samples (*n* = 91 and 49; Van Overwalle *et al.*, 2019c, 2020b, respectively). Perhaps more participants than the current study (*n* = 18–22) are needed to discern a clear pattern of positive and negative connectivity. Alternatively, the tasks in the earlier analyses involved familiar social scenes (cf. chocolate scenario from the introduction), of which the correct sequence is well-known, while the Belief SRT task is set in a somewhat artificial context, with a new and unfamiliar sequence to be learned implicitly or explicitly. As the level of awareness and knowledge of the sequence might vary greatly between participants, this may have resulted in this mixed pattern.

### Connectivity was not directly modulated by random sequences

As mentioned earlier, prior meta-analyses of SRT tasks (Hardwick *et al.*, 2013; Janacsek *et al.*, 2020) often compare the learned standard sequence against a random sequence or another baseline condition without a learned sequence. This reflects the assumption that a learned sequence rests on robust cerebellar involvement for encoding and automatizing, while novel or random sequences recruit a cerebellar process to a lesser extent. Surprisingly, this contrast did not reveal any activation of the cerebellum in the original Belief SRT studies (Ma *et al.*, 2021). Therefore, we could not include it in the current DCM analysis. Some differences in SRT designs may account for this lack of a random sequence effect. Perhaps most importantly, many earlier SRT tasks in neuroimaging studies presented a combination of standard and random sequences from the beginning (Hardwick *et al.*, 2013; Janacsek *et al.*, 2020), while the present Belief SRT studies began with a long Training phase of a standard sequence without any random disruption, as is typically done in behavioral research (Ma *et al.*, 2021). Although this set-up precluded an immediate examination of connectivity patterns in comparison with random sequences (in the Training phase), it showed significant contrasts against the later Test phase when random violations were introduced. It would be of theoretical interest for future studies to test how cerebello–cerebral connectivity is modulated by novel sequences against an already automatized sequence.

### Connectivity within the cerebrum and the cerebellum

Within the cerebrum, we found connections between the right and left TPJ, and connections between the right TPJ and the PCun during the initial Training phase. During the later Test phase, almost all cerebral ROIs (bilateral TPJ, bilateral TP and the caudate) were connected to each other, and most of these connections formed effectively closed loops during implicit learning. Although specific mentalizing areas are responsible for specific functions, our results suggest that these cerebral mentalizing areas exchange signals with each other in integrative functional synchrony, especially when participants were learning and automatizing a social sequence. The effective connections between the bilateral TPJ and the bilateral TP support previous studies, showing that these cerebral areas are co-activated during mentalizing (see meta-analyses by Van Overwalle and Baetens, 2009; Schurz *et al.*, 2014; Molenberghs *et al.*, 2016; Wang *et al.*, 2021). Also, while earlier studies on mentalizing showed effectively closed loops between the bilateral TPJ using DCM (Van Overwalle *et al.*, 2019c, 2020b), our DCM results further revealed effective connections between the TPJ and TP which are consistent with anatomical connections between the TPJ and TP via fiber tracts (Wang *et al.*, 2021).

Within the cerebellum, we found that most cerebellar ROIs were connected to each other within one hemisphere or across the two hemispheres, suggesting intra- and interhemispheric connectivity in the cerebellum. Our DCM findings are supported by DTI analyses of the human cerebellum which revealed short intracerebellar fibers representing a local intracerebellar circuitry (Catani *et al.*, 2008) as well as transverse white matter fibers crossing the two hemispheres at the level of the cerebellar vermis (Salamon *et al.*, 2007). It is likely that these paths are also used for exchanging social information.

### Implications

Studying the dynamic changes in activation of brain areas via connectivity is important to understand the functionality of the brain. In the current study, we showed robust effective cerebello–cerebral connectivity during the learning of belief sequences. However, functional and structural connectivity is not always a simple one-to-one mapping (Suárez *et al.*, 2020; Wang *et al.*, 2021). Therefore, it will be of interest to use structural techniques like DTI to test in more depth how the cerebello–cerebral relationships are organized via anatomical connections during implicit and explicit social sequence learning. This would provide even stronger evidence for the role of the posterior cerebellum in mentalizing with the sequential organization (Van Overwalle *et al.*, 2019b).

Also, for clinical populations with social deficits, it will be of interest for future studies to investigate whether there is a deficit within-cerebellar and cerebral mentalizing areas, or whether there is distorted connectivity between these mentalizing areas (Van Overwalle *et al.*, 2021a). For example, researchers showed that during the resting state, people with autism revealed no differences in TPJ organization and components compared to neurotypical participants, but they had a significant decrease in connectivity between the right TPJ and the left posterior cerebellar Crus II (Igelström *et al.*, 2017). As DCM can reveal the effective direction of connectivity, future studies can further reveal whether autistic patients have weak connections from the posterior cerebellum to the cerebral mentalizing areas or vice versa, resulting in their failure to flexibly learn and automatize sequential information during social cognition.

As the posterior cerebellum is close to the skull (Grimaldi *et al.*, 2016), our connectivity study suggests the posterior cerebellum as a possible new target for noninvasive brain stimulation (Cattaneo *et al.*, 2021; Van Overwalle *et al.*, 2021a). Previous studies showed that transcranial magnetic stimulation of the posterior cerebellum reduced response times for generating the correct order of mentalizing stories (Heleven *et al.*, 2021) and that transcranial direct current stimulation (tDCS) improved emotion discrimination (Ferrucci *et al.*, 2012). Together with our results of functional connectivity, future studies stimulating the posterior cerebellar Crus I/II and cortical areas, such as the TPJ, could shed light on the causal roles of cerebello–cerebral areas involved in social processes. Stimulating the cerebellum may also be possible indirectly. A study combining tDCS and fMRI showed that stimulating the prefrontal cortex not only induced stronger activation in this area but also resulted in increased cerebellar activation during implicit emotion regulation (Abend *et al.*, 2019). Future studies combining stimulation and fMRI are needed to test how functional connectivity is changed after stimulation. For example, during social sequence learning, it is of critical importance to see whether stimulating the posterior cerebellar Crus I/II can result in stronger TPJ activation and stronger connectivity with the cerebrum and so improves mentalizing capacities. Also vice versa, how stimulating cortical mentalizing areas might impact the posterior cerebellum and mentalizing functionality. Perhaps the combined stimulation of both cerebellar and cerebral areas might result in an additive effect, which might be advantageous for clinical purposes.

## Conclusion

Using DCM, we found closed loops between the posterior cerebellum (e.g. Crus I & II) and cerebral areas sensitive to mentalizing (i.e. TPJ, PCun and TP). The analysis suggests that a specific cerebello–cerebral mentalizing network is engaged while learning social belief sequences. The closed loops in this network confirm the hypothesis by (Van Overwalle *et al.* 2019a) that the posterior cerebellum receives signals from cerebral mentalizing areas about incoming social information and sends back confirmatory or corrective signals about the social sequences identified in this information to the same cerebral areas. Many unresolved questions remain and need to be answered in future studies: To what extent is awareness of a social sequence important in connectivity? Are there other sources that may lead to potential modulations which were quite rare in the present studies? How robust are patterns of positive downward and negative upward connectivity in social sequence learning revealed in prior DCM analyses? Although many questions remain and research on social sequence learning is still young, one fact is obvious: social belief understanding relies heavily on the connectivity of key mentalizing cerebral areas, such as the bilateral TPJ with the posterior cerebellum, and is therefore unlikely to function properly without the latter.

## Supplementary Material

nsac044_Supp

## References

[R1] Abend R., Sar-el R., Gonen T., et al. (2019). Modulating emotional experience using electrical stimulation of the medial-prefrontal cortex: a preliminary tDCS-fMRI study. *Neuromodulation*, 22(8), 884–93.doi: 10.1111/ner.12787. 29741803 PMC6226375

[R2] Buckner R.L., Krienen F.M., Castellanos A., Diaz J.C., Yeo B.T.T. (2011). The organization of the human cerebellum estimated by intrinsic functional connectivity. *Journal of Neurophysiology*, 106(5), 2322–45.doi: 10.1152/jn.00339.2011. 21795627 PMC3214121

[R3] Catani M., Jones D.K., Daly E., et al. (2008). Altered cerebellar feedback projections in Asperger syndrome. *NeuroImage*, 41(4), 1184–91.doi: 10.1016/j.neuroimage.2008.03.041. 18495494

[R4] Cattaneo Z., Ferrari C., Ciricugno A., et al. (2021). New horizons on non-invasive brain stimulation of the social and affective cerebellum. *Cerebellum*, 21, 482–96.doi: 10.1007/s12311-021-01300-4. 34270081

[R5] Cusack R., Papadakis N. (2002). New robust 3-D phase unwrapping algorithms: application to magnetic field mapping and undistorting echoplanar images. *NeuroImage*, 16(3), 754–64.doi: 10.1006/nimg.2002.1092. 12169259

[R6] Deroost N., Coomans D. (2018). Is sequence awareness mandatory for perceptual sequence learning: an assessment using a pure perceptual sequence learning design. *Acta Psychologica*, 183(January), 58–65.doi: 10.1016/j.actpsy.2018.01.002. 29331549

[R7] Diedrichsen J., King M., Hernandez-Castillo C., Sereno M., Ivry R.B. (2019). Universal transform or multiple functionality? Understanding the contribution of the human cerebellum across task domains. *Neuron*, 102(5), 918–28.doi: 10.1016/j.neuron.2019.04.021. 31170400 PMC6686189

[R8] Ferrucci R., Giannicola G., Rosa M., et al. (2012). Cerebellum and processing of negative facial emotions: cerebellar transcranial DC stimulation specifically enhances the emotional recognition of facial anger and sadness. *Cognition & Emotion*, 26(5), 786–99.doi: 10.1080/02699931.2011.619520. 22077643 PMC4234053

[R9] Friston K., Zeidman P., Litvak V. (2015). Empirical Bayes for DCM: a group inversion scheme. *Frontiers in Systems Neuroscience*, 9(November), 164.doi: 10.3389/fnsys.2015.00164. PMC4661273 26640432

[R10] Friston K.J., Harrison L., Penny W. (2003). Dynamic causal modelling. *NeuroImage*, 19(4), 1273–302.doi: 10.1016/S1053-8119(03)00202-7. 12948688

[R11] Friston K.J., Litvak V., Oswal A., et al. (2016). Bayesian model reduction and empirical Bayes for group (DCM) studies. *NeuroImage*, 128, 413–31.doi: 10.1016/j.neuroimage.2015.11.015. 26569570 PMC4767224

[R12] Frith C.D., Frith U. (2006). The neural basis of mentalizing. *Neuron*, 50(4), 531–4.doi: 10.1016/j.neuron.2006.05.001. 16701204

[R13] Geiger A., Cleeremans A., Bente G., Vogeley K. (2018). Social cues alter implicit motor learning in a serial reaction time task. *Frontiers in Human Neuroscience*, 12(May), 1–12.doi: 10.3389/fnhum.2018.00197. 29867420 PMC5960666

[R14] Glickstein M., May J.G., Mercier B.E. (1985). Corticopontine projection in the macaque: the distribution of labelled cortical cells after large injections of horseradish peroxidase in the pontine nuclei. *Journal of Comparative Neurology*, 235(3), 343–59.doi: 10.1002/cne.902350306. 3998215

[R15] Grimaldi G., Argyropoulos G.P., Bastian A., et al. (2016). Cerebellar transcranial direct current stimulation (ctDCS): a novel approach to understanding cerebellar function in health and disease. *The Neuroscientist*, 22(1), 83–97.doi: 10.1177/1073858414559409. 25406224 PMC4712385

[R16] Guell X., Gabrieli J.D.E., Schmahmann J.D. (2018). Triple representation of language, working memory, social and emotion processing in the cerebellum: convergent evidence from task and seed-based resting-state fMRI analyses in a single large cohort. *NeuroImage*, 172(January), 437–49.doi: 10.1016/j.neuroimage.2018.01.082. 29408539 PMC5910233

[R17] Hardwick R.M., Rottschy C., Miall R.C., Eickhoff S.B. (2013). A quantitative meta-analysis and review of motor learning in the human brain. *NeuroImage*, 67, 283–97.doi: 10.1016/j.neuroimage.2012.11.020. 23194819 PMC3555187

[R18] Heleven E., van Dun K., Van Overwalle F. (2019). The posterior cerebellum is involved in constructing social action sequences: an fMRI study. *Scientific Reports*, 9(1), 11110.doi: 10.1038/s41598-019-46962-7. PMC6668391 31366954

[R19] Heleven E., van Dun K., De Witte S., Baeken C., Van Overwalle F. (2021). The role of the cerebellum in social and non-social action sequences: a preliminary LF-rTMS study. *Frontiers in Human Neuroscience*, 15.doi: 10.3389/fnhum.2021.593821. PMC7947373 33716690

[R20] Hillebrandt H., Friston K.J., Blakemore S.J. (2014). Effective connectivity during animacy perception - dynamic causal modelling of human connectome project data. *Scientific Reports*, 4(1), 1–9.doi: 10.1038/srep06240. PMC4150124 25174814

[R21] Igelström K.M., Webb T.W., Graziano M.S.A. (2017). Functional connectivity between the temporoparietal cortex and cerebellum in autism spectrum disorder. *Cerebral Cortex*, 27(4), 2617–27.doi: 10.1093/cercor/bhw079. 27073219

[R22] Ito M. (2008). Control of mental activities by internal models in the cerebellum. *Nature Reviews Neuroscience*, 9(4), 304–13.doi: 10.1038/nrn2332. 18319727

[R23] Janacsek K., Shattuck K.F., Tagarelli K.M., Lum J.A.G., Turkeltaub P.E., Ullman M.T. (2020). Sequence learning in the human brain: a functional neuroanatomical meta-analysis of serial reaction time studies. *NeuroImage*, 207(May 2019), 116387.doi: 10.1016/j.neuroimage.2019.116387. 31765803

[R24] Karavasilis E., Christidi F., Velonakis G., et al. (2019). Ipsilateral and contralateral cerebro-cerebellar white matter connections: a diffusion tensor imaging study in healthy adults. *Journal of Neuroradiology*, 46(1), 52–60.doi: 10.1016/j.neurad.2018.07.004. 30098370

[R25] Kelly R.M., Strick P.L. (2003). Cerebellar loops with motor cortex and prefrontal cortex of a nonhuman primate. *Journal of Neuroscience*, 23(23), 8432–44.doi: 10.1523/jneurosci.23-23-08432.2003. 12968006 PMC6740694

[R26] Leggio M., Molinari M. (2015). Cerebellar sequencing: a trick for predicting the future. *The Cerebellum*, 14(1), 35–8.doi: 10.1007/s12311-014-0616-x. 25331541

[R27] Lewis P.A., Birch A., Hall A., Dunbar R.I.M. (2017). Higher order intentionality tasks are cognitively more demanding. *Social Cognitive and Affective Neuroscience*, 12(7), 1063–71.doi: 10.1093/scan/nsx034. 28338962 PMC5490680

[R28] Liebrand M., Karabanov A., Antonenko D., et al. (2020). Beneficial effects of cerebellar tDCS on motor learning are associated with altered putamen-cerebellar connectivity: a simultaneous tDCS-fMRI study. *NeuroImage*, 223(April), 117363.doi: 10.1016/j.neuroimage.2020.117363. 32919057

[R29] Ma Q., Pu M., Heleven E., et al. (2021). The posterior cerebellum supports implicit learning of social belief sequences. *Cognitive, Affective & Behavioral Neuroscience*, 21(5), 970–92.doi: 10.3758/s13415-021-00910-z. 34100254

[R0029a] Ma Q., Pu M., Haihambo N., Baetens K., et al. (2022). The posterior cerebellum and temporoparietal junction support explicit learning of social belief sequences. *Cognitive, Affective & Behavioral Neuroscience*.doi: 10.3758/s13415-021-00966-x. 34811709

[R30] Metoki A., Wang Y., Olson I.R. (2021). The social cerebellum: a large-scale investigation of functional and structural specificity and connectivity. *Cerebral Cortex*, 32, (5), 987–1003.doi: 10.1093/cercor/bhab260. PMC8890001 34428293

[R31] Molenberghs P., Johnson H., Henry J.D., Mattingley J.B. (2016). Understanding the minds of others: a neuroimaging meta-analysis. *Neuroscience and Biobehavioral Reviews*, 65, 276–91.doi: 10.1016/j.neubiorev.2016.03.020. 27073047

[R32] Morgan O.P., Slapik M.B., Iannuzzelli K.G., et al. (2021). The cerebellum and implicit sequencing: evidence from cerebellar ataxia. *The Cerebellum*, 20(2), 222–45.doi: 10.1007/s12311-020-01206-7. 33123963

[R33] Nissen M.J., Bullemer P. (1987). Attentional requirements of learning: evidence from performance measures. *Cognitive Psychology*, 19(1), 1–32.doi: 10.1016/0010-0285(87)90002-8.

[R34] Salamon N., Sicotte N., Drain A., et al. (2007). White matter fiber tractography and color mapping of the normal human cerebellum with diffusion tensor imaging. *Journal of Neuroradiology*, 34(2), 115–28.doi: 10.1016/j.neurad.2007.03.002. 17481730

[R35] Schilbach L., Bzdok D., Timmermans B., et al. (2012). Introspective minds: using ALE meta-analyses to study commonalities in the neural correlates of emotional processing, social & unconstrained cognition. *PLoS One*, 7(2), e30920.doi: 10.1371/journal.pone.0030920. PMC3272038 22319593

[R36] Schmahmann J.D. (1996). From movement to thought: anatomic substrates of the cerebellar contribution to cognitive processing. *Human Brain Mapping*, 4(3), 174–98.doi: 10.1002/(SICI)1097-0193(1996)4:3<174::AID-HBM3>3.0.CO;2-0. 20408197

[R37] Schurz M., Aichhorn M., Martin A., Perner J. (2013). Common brain areas engaged in false belief reasoning and visual perspective taking: a meta-analysis of functional brain imaging studies. *Frontiers in Human Neuroscience*, 7(NOV), 712.doi: 10.3389/fnhum.2013.00712. PMC3814428 24198773

[R38] Schurz M., Radua J., Aichhorn M., Richlan F., Perner J. (2014). Fractionating theory of mind: a meta-analysis of functional brain imaging studies. *Neuroscience and Biobehavioral Reviews*, 42, 9–34.doi: 10.1016/j.neubiorev.2014.01.009. 24486722

[R39] Sokolov A.A., Erb M., Grodd W., Pavlova M.A. (2014). Structural loop between the cerebellum and the superior temporal sulcus: evidence from diffusion tensor imaging. *Cerebral Cortex*, 24(3), 626–32.doi: 10.1093/cercor/bhs346. 23169930

[R40] Suárez L.E., Markello R.D., Betzel R.F., Misic B. (2020). Linking structure and function in macroscale brain networks. *Trends in Cognitive Sciences*, 24(4), 302–15.doi: 10.1016/j.tics.2020.01.008. 32160567

[R41] Suzuki L., Coulon P., Sabel-Goedknegt E.H., Ruigrok T.J.H. (2012). Organization of cerebral projections to identified cerebellar zones in the posterior cerebellum of the rat. *Journal of Neuroscience*, 32(32), 10854–69.doi: 10.1523/JNEUROSCI.0857-12.2012. 22875920 PMC6621006

[R42] Taylor J.A., Klemfuss N.M., Ivry R.B. (2010). An explicit strategy prevails when the cerebellum fails to compute movement errors. *The Cerebellum*, 9(4), 580–6.doi: 10.1007/s12311-010-0201-x. 20697860 PMC2996538

[R43] Tzvi E., Stoldt A., Witt K., Krämer U.M. (2015). Striatal–cerebellar networks mediate consolidation in a motor sequence learning task: an fMRI study using dynamic causal modelling. *NeuroImage*, 122, 52–64.doi: 10.1016/j.neuroimage.2015.07.077. 26244275

[R44] Van Overwalle F. (2009). Social cognition and the brain: a meta-analysis. *Human Brain Mapping*, 30(3), 829–58.doi: 10.1002/hbm.20547. 18381770 PMC6870808

[R45] Van Overwalle F., Baetens K., Mariën P., Vandekerckhove M. (2014). Social cognition and the cerebellum: a meta-analysis of over 350 fMRI studies. *NeuroImage*, 86, 554–72.doi: 10.1016/j.neuroimage.2013.09.033. 24076206

[R46] Van Overwalle F., De Coninck S., Heleven E., et al. (2019a). The role of the cerebellum in reconstructing social action sequences: a pilot study. *Social Cognitive and Affective Neuroscience*, 14(5), 549–58.doi: 10.1093/scan/nsz032. 31037308 PMC6545532

[R47] Van Overwalle F., Manto M., Leggio M., Delgado-García J.M.J.M. (2019b). The sequencing process generated by the cerebellum crucially contributes to social interactions. *Medical Hypotheses*, 128(February), 33–42.doi: 10.1016/j.mehy.2019.05.014. 31203906

[R48] Van Overwalle F., Van de Steen F., Mariën P. (2019c). Dynamic causal modeling of the effective connectivity between the cerebrum and cerebellum in social mentalizing across five studies. *Cognitive, Affective & Behavioral Neuroscience*, 19(1), 211–23.doi: 10.3758/s13415-018-00659-y. 30361864

[R49] Van Overwalle F., Ma Q., Heleven E. (2020a). The posterior crus II cerebellum is specialized for social mentalizing and emotional self-experiences: a meta-analysis. *Social Cognitive and Affective Neuroscience*, 15(9), 905–28.doi: 10.1093/scan/nsaa124. 32888303 PMC7851889

[R50] Van Overwalle F., Van de Steen F., van Dun K., Heleven E. (2020b). Connectivity between the cerebrum and cerebellum during social and non-social sequencing using dynamic causal modelling. *NeuroImage*, 206, 116326.doi: 10.1016/j.neuroimage.2019.116326. 31678499

[R51] Van Overwalle F., Baeken C., Campanella S., et al. (2021a). The role of the posterior cerebellum in dysfunctional social sequencing. *The Cerebellum*, (0123456789).doi: 10.1007/s12311-021-01330-y. 34637054

[R52] Van Overwalle F., Pu M., Ma Q., et al. (2021b). The involvement of the posterior cerebellum in reconstructing and predicting social action sequences. *The Cerebellum*, (0123456789).doi: 10.1007/s12311-021-01333-9. 34694590

[R53] Van Overwalle F., Baetens K. (2009). Understanding others’ actions and goals by mirror and mentalizing systems: a meta-analysis. *NeuroImage*, 48(3), 564–84.doi: 10.1016/j.neuroimage.2009.06.009. 19524046

[R54] Wang Y., Metoki A., Xia Y., Zang Y., He Y., Olson I.R. (2021). A large-scale structural and functional connectome of social mentalizing. *NeuroImage*, 236(December2020), 118115.doi: 10.1016/j.neuroimage.2021.118115. PMC12317760 33933599

[R55] Zeidman P., Jafarian A., Corbin N., et al. (2019). A guide to group effective connectivity analysis, part 1: first level analysis with DCM for fMRI. *NeuroImage*, 200(June), 174–90.doi: 10.1016/j.neuroimage.2019.06.031. 31226497 PMC6711459

[R56] Zhou Y., Liang M., Tian L., et al. (2007). Functional disintegration in paranoid schizophrenia using resting-state fMRI. *Schizophrenia Research*, 97(1–3), 194–205.doi: 10.1016/j.schres.2007.05.029. 17628434

[R57] Zhou Y., Zeidman P., Wu S., et al. (2018). Altered intrinsic and extrinsic connectivity in schizophrenia. *NeuroImage: Clinical*, 17(June2017), 704–16.doi: 10.1016/j.nicl.2017.12.006. 29264112 PMC5726753

