# Supplementary material for: Effective cerebello–cerebral connectivity during implicit and explicit social belief sequence learning using dynamic causal modeling
Source: Soc Cogn Affect Neurosci. 2022 Jul 7;18(1):nsac044. doi: 10.1093/scan/nsac044 (PMC9951265; doi:10.1093/scan/nsac044)
Supplement: nsac044_Supp [file nsac044_supp.zip › scan-22-071-File006.pdf]

Supplementary materials for

Connectivity between the cerebrum and cerebellum during implicit and explicit social belief sequence learning using dynamic causal modelling

Qianying Ma[1], Min Pu[1], Naem P. Haihambo[1], Kris Baetens[1], Elien Heleven[1], Natacha Deroost[1], Chris Baeken[2,3,4,5], Frank Van Overwalle[1]

1. Department of Psychology, Center for Neuroscience, Vrije Universiteit Brussel, Belgium
2. Faculty of Medicine and Health Sciences, Department of Head and Skin, Ghent Experimental
3. Psychiatry (GHEP) lab, Ghent University, Ghent, Belgium
4. Department of Psychiatry, University Hospital (UZBrussel), Brussels, Belgium
5. Eindhoven University of Technology, Department of Electrical Engineering, Eindhoven, the Netherlands

Standard and Random sequences

Table S1: The Standard sequence and pseudo-random sequences of the Random Orientation.

Standard Sequence to be learned (repeated every 1 – 16 trials)																
Location	1	1	4	3	4	3	2	2	2	1	1	4	3	4	3	2
Protagonist	M	M	Fe	M	Fe	M	Fe	Fe	Fe	Fe	Fe	M	Fe	M	Fe	M
Belief	T	Fa	T	T	Fa	Fa	T	Fa	Fa	T	Fa	T	T	Fa	Fa	T
Prior true trial		-1			-2	-2		-1	-2		-1			-2	-2	
Random Orientation (repeated every 1 – 32 trials)																
Location	1	1	4	3	4	3	2	2	2	1	1	4	3	4	3	2
Protagonist	M	M	Fe	M	Fe	M	Fe	Fe	Fe	Fe	Fe	M	Fe	M	Fe	M
Orientation	T	T	T	T	Fa	Fa	T	Fa	T	T	T	T	T	Fa	Fa	T
Prior true trial					-2	-2		-1						-2	-2	
(continued: trials 17 - 32)																
Location	1	1	4	3	4	3	2	2	2	1	1	4	3	4	3	2
Protagonist	M	M	Fe	M	Fe	M	Fe	Fe	Fe	Fe	Fe	M	Fe	M	Fe	M
Orientation	T	T	T	T	Fa	Fa	T	T	Fa	T	Fa	T	T	Fa	Fa	T
Prior true trial					-2	-2			-1		-1			-2	-2	
Total Random																
All dimensions were totally randomized with the limitation of at most 2 subsequent trials of the same true or false type, consistent with the Standard blocks																

Note: In the Belief SRT task: M = male, Fe = female, T = true, Fa = false. In the Control SRT task: Male = Square, Female = Circle, True = Blue/Green, False = Orange/Black. Random sequences were made two times as long to increase unpredictability. Location = location on the screen from left to right, Protagonist = Papa Smurf & Smurfette (Belief SRT task), and colored Shape (Control SRT task), Orientation = Belief Orientation (Belief SRT task) and Shape Color Orientation (Control SRT task).

Because the previous behavioral study (Ma, Heleven, et al., 2021) did not reveal sequence learning about protagonists and the flowers’ locations, sequences tied to protagonists´ or flowers´ locations were not used to test random violations in the previous neuroimaging studies (Ma, Pu, Haihambo, et al., 2021; Ma, Pu, Heleven, et al., 2021).

Results of additional DCMs.

We ran additional DCM analyses to rule out the possibility of multicollinearity as the cause of connection patterns showing mixed valence.

Reduced model 1

In this model, we included only the hypothesized cerebellar and cerebral mentalizing areas (i.e., the left Crus I, the bilateral Crus II and the bilateral TPJ) for the implicit and explicit Belief SRT tasks. As shown in **Table S2-S3**, positive or negative signs are largely consistent with the original model.

Table S2 Averaged connections in units of 1/s (Hz) for the contrast Standard block at Training > Standard block at Test for reduced model 1.

from<br>to	L Crus I	L Crus II	R Crus II	L TPJ	R TPJ
**Implicit Belief SRT task**					
	Fixed connectivity				
L Crus I	-0.47**	0.19**	-0.10*		0.14**
L Crus II	0.19**	-0.35**	0.16**		
R Crus II	-0.12*	0.16**	-0.72**	0.04*	-0.08*
L TPJ	0.14**		0.15**	-0.33**	
R TPJ				0.12*	-0.54**
	Modulatory connectivity				
L Crus I	-0.03	0.01	0.03	-0.02	-0.02
L Crus II		-0.34*	-0.03		-0.01
R Crus II	0.01	0.03	-0.24*	0.03	0.01
L TPJ	0.02	-0.03	-0.05	-0.47*	0.01
R TPJ	0.04		0.01		-0.04
**Explicit Belief SRT task**					
	Fixed connectivity				
L Crus I	-0.33**	0.33**		0.10**	-0.06*
L Crus II	0.18**	-0.45**	-0.04*	0.12**	
R Crus II	0.07**		-0.46**	0.15**	-0.12**
L TPJ	-0.04*	0.09**		-0.49**	
R TPJ	-0.04*	0.10**		0.09**	-0.56**
	Modulatory connectivity				
L Crus I	-1.00**	-0.28*	0.01	0.01	-0.01
L Crus II	-0.07	-0.69**		-0.07	-0.02
R Crus II	-0.19*	0.21*	-0.90**	0.03	-0.04
L TPJ	-0.02	0.01	-0.01	-0.62**	0.02
R TPJ	-0.02	-0.02	0.03	-0.19*	-0.80**

Note: Table shows value of estimates (empty cells denote estimate = 0). Cell entries refer to connections from top row ROIs to left column ROIs with posterior probability ** p > .95, * p > .50. Bold denotes closed loops. *Italic* denotes self-inhibitions in diagonal cells. Red color indicates different signs of estimates compared to the original model in the article. Light gray denotes cerebellar-cortical connectivity. L = Left, R = Right, TPJ = Temporoparietal Junction.

Table S3 Averaged connections in units of 1/s (Hz) for the contrast Standard block at Test > Standard block at Training for reduced model 1.

to \ from	L Crus I	L Crus II	R Crus II	L TPJ	R TPJ
**Implicit Belief SRT task**					
	Fixed connectivity				
L Crus I	*-0.61***	0.18**	**-0.21****	**0.06***	
L Crus II		*-0.70***	**-0.20****	**0.12****	**0.15****
R Crus II	**-0.21****	**0.18****	*-0.30***	**0.12****	**0.06***
L TPJ	**-0.12****	**0.18****	**-0.13****	*-0.64***	**0.25****
R TPJ		-0.05*	**-0.05***	**0.11****	*-0.53***
	Modulatory connectivity				
L Crus I	*-0.03*	-0.05	0.01	-0.02	0.04
L Crus II	-0.04	*-0.36**	0.03	-0.41**	0.04
R Crus II		-0.01	*-0.02*	-0.20*	0.03
L TPJ	-0.01	-0.02	-0.01	*-0.03*	-0.01
R TPJ	-0.05		0.04	-0.21*	*-0.48**
**Explicit Belief SRT task**					
	Fixed connectivity				
L Crus I	*-0.46***	0.25**	-0.17**	**0.32****	**-0.15****
L Crus II		*-0.40*	**0.06***		0.19**
R Crus II		**0.12****	*-0.58***	**0.07***	
L TPJ	**0.18****		**-0.21****	*-0.47***	**0.26****
R TPJ	**-0.25****		0.06*	**0.20****	*-0.71***
	Modulatory connectivity				
L Crus I	*-0.03*	-0.04	-0.04	-0.05	0.05
L Crus II	-0.03	*-0.70***	-0.30*	0.01	0.01
R Crus II	-0.06	-0.02	*-0.02*		0.03
L TPJ	-0.21*	-0.03	-0.04	*-0.85***	-0.03
R TPJ	-0.01		-0.03	-0.02	

Note: Table shows value of estimates (empty cells denote estimate = 0). Cell entries refer to connections from top row ROIs to left column ROIs with posterior probability ** p > .95, * p > .50. Bold denotes closed loops. *Italic* denotes self-inhibitions in diagonal cells. Red color indicates different signs of estimates compared to the original model in the article. Light gray denotes cerebellar-cortical connectivity. L = Left, R = Right, TPJ = Temporoparietal Junction.

Reduced model 2

In model, we only included robustly significant ROIs for each of the learning phases. As shown in **Table S4-S5**, positive or negative signs are largely consistent with the original model.

Table S4 Averaged connections in units of 1/s (Hz) for the contrast Standard block at Training > Standard block at Test for reduced model 2.

to \ from	L Crus I	L Lob. VI	R Lob. VI	Precuneus	L TPJ	R TPJ
**Implicit Belief SRT task**						
	Fixed connectivity					
L Crus I	_-0.46**_	__0.08**__		-0.08**	__0.12**__	__0.08**__
L Lob. VI	__0.12**__	_-0.66**_	0.12**	__-0.05*__	0.10**	__-0.20**__
R Lob. VI	-0.06**		_-0.76**_			
Precuneus		__0.04*__		_-0.57**_		
L TPJ	__0.19**__		-0.09**		_-0.50**_	
R TPJ	__0.43**__	__0.03*__	-0.18**	0.10**	-0.16**	_-0.42**_
	Modulatory connectivity					
L Crus I	-1.20**	-0.03		0.18*	-0.01	__-0.38**__
L Lob. VI	-0.02	-0.61**	-0.23*	0.04		-0.01
R Lob. VI		0.06	-0.02	0.01	0.04	0.03
Precuneus	-0.05	-0.01	-0.02	-0.64**	0.02	-0.04
L TPJ	-0.05	0.04	0.03	0.03	-0.35**	-0.01
R TPJ	__-0.39**__	0.02	0.30*	-0.03	0.02	-0.03
**Explicit Belief SRT task**						
	Fixed connectivity					
L Crus I	_-0.70**_		__0.14**__		__0.10**__	__-0.09**__
L Lob. VI		_-0.59**_	__-0.17**__	0.26**		__0.15**__
R Lob. VI	__0.09**__	__-0.07**__	_-0.48**_	0.29**		0.03*
Precuneus				_-0.72**_		__0.19**__
L TPJ	__0.03*__	0.10**	-0.03*		_-0.64**_	
R TPJ	__0.03*__	__-0.10**__		__0.22**__		_-0.49**_
	Modulatory connectivity					
L Crus I	_-0.03_	0.02	-0.03		-0.04	0.26*
L Lob. VI	-0.04	_-0.22*_	0.05	-0.02	-0.03	-0.04
R Lob. VI	-0.07	0.01	_-0.69**_	-0.05	-0.16*	-0.04
Precuneus	-0.02	-0.04	0.01	_-0.74**_	-0.29*	-0.02
L TPJ	-0.05	-0.05	0.07	0.01	_-0.04_	0.06
R TPJ	-0.05	0.00	0.01	-0.03	-0.03	_-0.76**_

Note: Table shows value of estimates (empty cells denote estimate = 0). Cell entries refer to connections from top row ROIs to left column ROIs with posterior probability ** $p > .95$, * $p > .50$. Bold denotes closed loops. *Italic* denotes self-inhibitions in diagonal cells. Red color indicates different signs of estimates compared to the original model in the article. Light gray denotes cerebellar-cortical connectivity. L = Left, R = Right, Lob. VI = Cerebellar lobule VI; TPJ = Temporoparietal Junction.

Table S5 Averaged connections in units of 1/s (Hz) for the contrast Standard block at Test > Standard block at Training for reduced model 2.

from<br>to	L Crus II	R Crus II	L TP	R TP	L Caudate
**Implicit Belief SRT task**					
	Fixed connectivity				
L Crus II		-0.06*		0.22**	
R Crus II		*-0.11**	0.05*		0.18**
L TP	0.08*		*-0.06**		
R TP			0.07*	*-0.19***	
L Caudate					*-0.20***
	Modulatory connectivity				
L Crus II	*-0.66***	-0.33**	-0.46**	0.01	0.01
R Crus II	-0.01	*0.03*	-0.19*	-0.02	-0.02
L TP	0.01	-0.49**	*-0.01*	0.02	-0.01
R TP		-0.22*	-0.01	*-0.02*	-0.01
L Caudate		-0.42**	-0.03	0.01	*-0.79***
**Explicit Belief SRT task**					
	Fixed connectivity				
L Crus II	*-0.55***	**0.16****	**-0.09***	**-0.17****	**0.17****
R Crus II	**-0.04***	*-0.30***		**0.15****	**0.08***
L TP	**-0.19****		*-0.32***	**0.22****	**0.18****
R TP	**-0.23****	**0.21****	**-0.12****	*-0.58***	**0.14****
L Caudate	**-0.13****		**0.10***	**0.12***	*-0.64***
	Modulatory connectivity				
L Crus II		-0.06	-0.01	-0.03	0.03
R Crus II	-0.04	*-0.47**	-0.06	-0.40*	0.02
L TP	-0.01	-0.07	*-0.01*	-0.07	-0.01
R TP	0.04	-0.28*	-0.03	*-0.02*	0.02
L Caudate	-0.03	-0.04	-0.06	-0.01	*-0.85**

Note: Table shows value of estimates (empty cells denote estimate = 0). Cell entries refer to connections from top row ROIs to left column ROIs with posterior probability ** p > .95, * p > .50. Bold denotes closed loops. *Italic* denotes self-inhibitions in diagonal cells. Red color indicates different signs of estimates compared to the original model in the article. Light gray denotes cerebellar-cortical connectivity. L = Left, R = Right, TP = Temporal Pole, TPJ = Temporoparietal Junction.
